# A Soldering Iron Safety State Detection Method Based on Instance-Level Interaction Understanding

**DOI:** 10.3390/s26134238

**Published:** 2026-07-03

**Authors:** Zhenqian Shen, Runkun Xu, Peipei Zhang, Zhibin Jiang, Zijing Zhang

**Affiliations:** 1 School of Electronic and Information Engineering, Tiangong University, Tianjin 300387, China; shenzhenqian@tiangong.edu.cn (Z.S.); 2430080945@tiangong.edu.cn (R.X.); 15128922239@163.com (P.Z.); 2 Hangzhou Hikvision Robotics Technology Co., Ltd., Hangzhou 310052, China; jiangzhibin@hikrobotics.com

**Keywords:** soldering iron safety, visual safety monitoring, interaction state recognition, relation-aware modeling, SISID

## Abstract

In electronic training scenarios, the safety risk of a soldering iron cannot be determined by object detection alone, as its state must be further distinguished among hand-held, stand-supported, desk-exposed, and uncertain interactions. To address this problem, this paper proposes RISNet, the Relation-aware Interaction State Network, which establishes a two-stage instance-level interaction understanding framework for soldering iron safety monitoring. In the first stage, YOLO is used to generate candidate instances of soldering irons and related environmental objects, and dual-layer feature fusion is adopted to jointly exploit shallow details and deep semantics. In the second stage, the soldering iron is treated as the interaction subject. The Pointer-Head models associations between the subject and contextual objects, and the State-Head predicts the safety state conditioned on subject-object relational constraints. To reduce false alarms from false detections and weak interactions, RISNet introduces a Quality-Head that estimates the reliability of each interaction conclusion and filters low-quality predictions during inference. The unknown label is used during training as conservative supervision for weak, unreliable, or indeterminate interaction evidence, with semantics close to the no-interaction label in HOI. This paper also constructs the Soldering Iron Safety Interaction Dataset (SISID) to support detection, interaction modeling, and state evaluation of slender metallic tools in training scenarios. On the SISID validation split, RISNet achieves an Overall F1 of 95.38%, an Overall Precision of 96.73%, and an inference speed of 57.1 FPS, satisfying the centralized single-frame polling requirement considered in this work.

## 1. Introduction

Soldering irons are among the most frequently used basic tools in university electronic training, vocational skills training, and entry-level industrial training, while also posing potential burn risks. Existing studies on soldering education have shown that beginners are prone to burns, soldering failures, and other operational problems, whereas instructors must simultaneously demonstrate procedures, inspect learners, and correct errors, making continuous and fine-grained supervision of each learner difficult [1]. Consistently, studies on soldering training process analysis have also indicated that when the number of trainees is large and their skill levels vary substantially, relying solely on instructor experience increases the guidance burden and makes it difficult to form stable and quantitative evaluations of hazardous actions and operation quality [2]. Recent studies on manufacturing teaching laboratories further suggest that real-time visual detection for personal protective equipment compliance and experimental process safety is becoming an important technical direction for safety management in teaching scenarios [3]. Therefore, constructing a visual monitoring method that can automatically perceive soldering iron states and identify potential risks in training scenarios has direct value for safety management and also aligns with the development of intelligent educational equipment and digital laboratory supervision.

Soldering iron safety interaction state recognition goes beyond the existence recognition of a soldering iron in the conventional object detection sense. For practical safety supervision, the system must detect the soldering iron and further understand whether it is safely held, properly placed on a stand, or exposed on a desk and detached from safe support, which requires intervention. Recent safety monitoring studies have shown that judgments based only on general rules or static object presence often have difficulty adapting to complex operational contexts, thereby affecting the reliability of violation detection. Introducing an understanding of action semantics or interaction relations can make safety state recognition more robust [4]. Similarly, in other safety-critical tool scenarios, researchers have started to shift from pure object localization to the definition and recognition of risk states, showing that whether a tool is in a hazardous state has greater practical significance for supervision than whether the tool is detected [5,6]. This issue is particularly prominent in soldering iron monitoring because the same soldering iron may have completely different safety implications under different interaction contexts.

Single-stage object detectors represented by YOLO have been widely applied in industrial vision scenarios because of their compact structures and fast inference speed [7,8,9]. However, studies on small-object detection under complex backgrounds have repeatedly shown that a single deep semantic feature often cannot simultaneously preserve local edge details and robust representations. Adaptive cross-layer fusion and multi-scale enhancement remain key approaches for improving detection stability [10,11,12]. For soldering iron detection in training scenarios, this challenge involves both stable target detection and correct relational context understanding. Our preliminary results with direct state detection suggest that appearance- and location-driven modeling may confuse visually exposed but safe cases, such as hand-held or stand-supported soldering irons, with violation states. At the same time, desk clutter, slender similar objects, metallic reflections, and co-occurring background textures further amplify the problem of false positives. Therefore, the key challenge of this task includes missed detection and the suppression of false alarms caused by insufficient relational understanding under real-time constraints.

Human–Object Interaction (HOI) detection provides inspiration for understanding such state-dependent tasks. HOI Transformer, HOTR, and related methods have shown that interaction recognition benefits from joint modeling of subjects, objects, and their relations with global context [13,14]. By explicitly associating humans and objects and modeling high-level semantic dependencies, these methods show strong potential for complex scene understanding. Related studies also indicate that although general HOI frameworks have strong relation representation capabilities, two-stage exhaustive pairing, complex decoding structures, and post-processing procedures introduce additional inference costs, which limits their adaptability to low-latency deployment scenarios [15,16]. For soldering iron safety interaction state recognition in training scenarios, this means that the system must introduce relational semantics between the subject and the environment while controlling the additional computational overhead caused by interaction modeling.

Under practical deployment conditions, the system adopts centralized polling over a large number of cameras and uses sampled single-frame images from the polling stream. Since the interval between two observations from the same camera varies with system load, stable inter-frame relations are difficult to obtain. Therefore, this paper formulates soldering iron safety state detection as an instance-level interaction understanding problem in single-frame images, aiming to provide an engineering-feasible visual solution for real-time tool-level safety monitoring in electronic training scenarios. In summary, the main contributions of this work are as follows:

1. We propose RISNet, a two-stage instance-level interaction understanding framework for soldering iron safety interaction state recognition in training scenarios. The framework builds state recognition on the joint modeling of the subject, objects, and context, thereby addressing the relation-dependent nature of tool-state safety monitoring.
2. We design a compact interaction pipeline consisting of dual-layer feature fusion, Pointer-Head, State-Head, and Quality-Head. Dual-layer feature fusion enhances instance representations of slender small objects. Pointer-Head establishes key associations between the soldering iron subject and environmental objects. State-Head performs state prediction under joint subject–context conditions. Quality-Head estimates interaction reliability and filters low-quality conclusions during inference.
3. We construct the Soldering Iron Safety Interaction Dataset (SISID) and conduct validation-set experiments under the current dataset protocol. The experimental results show that the proposed method achieves a favorable balance among overall performance, recognition of the desk-exposed state, false-alarm control, and the processing requirement of centralized single-frame polling.

## 2. Related Work

### 2.1. Visual Safety Monitoring in Laboratories and Training Environments

Research on intelligent safety supervision for laboratories and training scenarios initially focused mainly on environmental parameter perception, networked early-warning systems, and basic monitoring systems [17]. In recent years, it has gradually expanded toward visual perception of human operations and tool-use processes. Guo et al. [17] noted in chemical laboratory operation monitoring that experimental safety and result reproducibility depend strongly on standardized operating procedures, while traditional manual inspection and retrospective evaluation are labor-intensive and difficult to scale. Meanwhile, complex human–tool interactions and temporal dependencies between procedural steps make it difficult for methods based only on static poses or frame-level detection to stably identify operational deviations [18]. Liu et al. further combined YOLO detection with multi-object tracking and introduced laboratory risk perception and responsibility traceability, showing that visual methods can support a management workflow spanning anomaly detection, subject localization, and process review [19]. In addition, video-analysis studies on abnormal laboratory behavior indicate that visual supervision frameworks combining human keypoints, pose estimation, and behavior recognition are promoting a shift in laboratory safety management from static object recognition to dynamic behavior understanding [20]. These studies show that the core requirement of laboratory safety monitoring has moved from coarse scene perception toward fine-grained recognition of frequent risk behaviors, operating subjects, and specific work processes.

This trend is more direct in studies related to soldering and soldering iron training. Yasunaga et al. proposed a soldering danger detection system based on object detection, hand tracking, and pose estimation, in which the soldering iron tip is localized and the spatial position of fingers is used to judge dangerous use states, demonstrating the feasibility of visual perception for soldering iron safety assistance [1]. From the perspective of training evaluation, Toyoshima et al. used haptic interaction and LSTM to quantitatively analyze soldering motion trajectories and pointed out that instructor experience alone has difficulty supporting both safety supervision and skill evaluation when multiple learners are trained in parallel [2]. Meanwhile, studies on state detection for other high-risk tools also show that automated systems are increasingly concerned with whether a tool is in a dangerous or activated state, which further strengthens the necessity of state-level perception in safety supervision [5]. Overall, existing training safety studies have confirmed the practical value of this task. However, most studies still focus separately on action standardization, responsibility traceability, or local dangerous-contact judgment. Visual discrimination of whether the same tool constitutes a violation under different interaction contexts remains insufficiently discussed, and a unified and efficient visual modeling framework is still lacking.

### 2.2. YOLO-Based Real-Time Object Detection and State Recognition

The YOLO series has established single-stage object detection as a mainstream approach for real-time visual tasks. YOLO integrates object localization and category prediction into a unified end-to-end detection framework, providing an efficient foundation for real-time scene understanding [7]. YOLOv3 further achieved a more practical balance between detection performance and speed [8]. YOLOX continued to optimize detection-head design and training strategies for engineering usability, demonstrating the sustained applicability of single-stage frameworks in industrial deployment [9]. In industrial vision tasks related to electronic manufacturing and soldering, YOLO-based models have also been applied to PCB electronic component soldering defect detection, further confirming the practical value of this route in engineering deployment [21]. Accordingly, YOLO-based methods are widely used in safety monitoring, object inspection, and laboratory scene perception tasks [19,21].

However, the advantages of YOLO frameworks mainly lie in fast determination of whether a target exists and where it is located. When the task involves small objects, complex backgrounds, and state-sensitive scenarios, the limitations of single-stage detection become more evident. Studies on small-object detection under complex backgrounds have shown that a single deep semantic feature often cannot simultaneously preserve local edge details and robust representations. Therefore, shallow local information and cross-layer feature fusion are needed to alleviate detection difficulty [10,11,12]. Adaptive feature fusion studies further point out that small objects in natural or complex scenes are prone to missed detections due to detail loss and false detections due to background interference, requiring learnable cross-layer fusion to improve the collaborative representation of multi-scale features [11]. This observation is directly relevant to soldering iron scenarios: the soldering iron tip is thin and small, and it often co-occurs with desktop devices, wires, reflective metals, and slender clutter. Relying only on single-layer deep semantic features may lead to false detections or unstable localization. More importantly, even when the detection box can stably cover the soldering iron body, the output of a single-stage detector usually lacks the relational semantics needed to describe whether the soldering iron is safely held, supported by a stand, or exposed in a hazardous area on the desk. Therefore, directly applying a conventional YOLO detection paradigm to soldering iron safety interaction state recognition may produce an imbalance in which detection recall is high while violation judgments introduce many false alarms.

### 2.3. Human–Object Interaction and Relation Modeling

Human–Object Interaction detection provides a mature research basis for relation-aware visual understanding. Representative transformer-based methods such as HOI Transformer and HOTR model humans, objects, and interaction categories jointly, showing that explicit subject–object association is important for recognizing interaction semantics in complex scenes [13,14]. Subsequent studies further improve relation modeling through global context, query-based decoding, and structured matching strategies [15,16]. These methods demonstrate strong general interaction representation ability and provide useful inspiration for the design of Pointer-Head and context-conditioned state prediction in RISNet.

The soldering iron safety task studied in this paper has a narrower and more engineering-oriented setting than standard HOI detection. The subject category is fixed as the soldering iron, the interaction objects are limited to hands, stands, and desks, and the output is a fixed safety-state label. In addition, the deployed system uses centralized polling over many cameras and processes sampled single-frame images under latency constraints. Directly adopting a full HOI transformer pipeline would introduce a broader decoding space and additional implementation costs for a task with a small and fixed relation vocabulary. RISNet therefore follows the relation-aware motivation of HOI methods while using a compact two-stage design tailored to soldering iron state recognition and false-alarm control.

### 2.4. Summary and Discussion of Existing Studies

Overall, existing laboratory safety monitoring studies have demonstrated the potential of visual methods in risk perception, process supervision, and training assistance [1,2,3,5,17,18,19,20]. YOLO-series detectors provide a mature and efficient technical foundation for real-time deployment [7,8,9,10,11,12,21], and HOI studies offer useful principles for subject–object relation modeling [13,14,15,16]. These studies provide an important foundation for automatic perception in training scenarios, while several specific challenges remain when they are applied to soldering iron safety interaction state recognition.

Many existing methods focus on whether a target appears, whether an action occurs, or whether equipment is compliant, while the semantics of tool states remain insufficiently modeled. Soldering irons have slender, small-scale visual characteristics and are easily affected by background interference. In complex desktop scenes, locally similar objects and metallic reflections can cause false detections or incorrect state judgments. Soldering iron safety state detection depends on the appearance of the subject and on the relations between the subject and environmental entities such as hands, stands, and desks. Therefore, a more targeted, lightweight interaction modeling mechanism is needed under real-time constraints. Based on this motivation, this paper proposes RISNet to address complex logical state understanding and false-alarm control in soldering iron safety interaction state recognition.

## 3. Materials and Methods

### 3.1. Task Definition and Overall Framework

**Figure 1:**
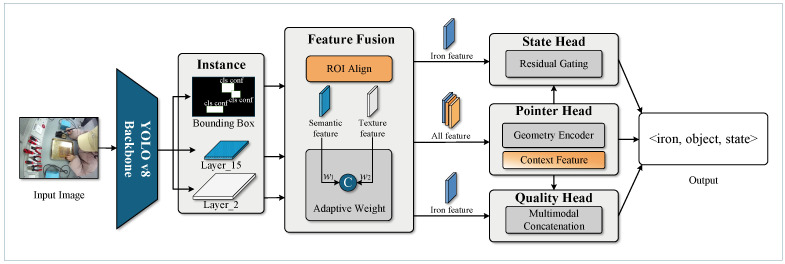
Overall framework of the proposed RISNet. Different colors denote different functional modules.

This paper proposes RISNet as a two-stage instance-level interaction understanding framework for soldering iron safety interaction state recognition in training scenarios. The overall pipeline first uses a YOLOv8 detector to generate candidate instances and extract features, then performs interaction reasoning with dual-layer feature fusion, Pointer-Head, State-Head, and Quality-Head. In this framework, the soldering iron is treated as the subject, the most relevant interaction object is selected from the remaining candidates, and state prediction is refined by an additional reliability check. The overall workflow is shown in Figure 1.

Let the input image be denoted as *I*. The first-stage instance detector outputs a candidate set B, where each candidate consists of a bounding box, a category, and a detection confidence score. The candidate categories are limited to desk, hand, stand, and iron.

$$B={\left\{\left(b_{i},c_{i},p_{i}\right)\right\}}_{i=1}^{N},\;c_{i}\in \left\{\mathrm{desk},\mathrm{hand},\mathrm{stand},\mathrm{iron}\right\}$$

In this expression, $b_{i}\in R^{4}$ is the *i*-th candidate box, $c_{i}$ is the candidate category, $p_{i}$ is the detector confidence score, and *N* is the number of candidate instances in the current image. This representation describes only instance-level candidates and does not contain interaction semantics.

For any soldering iron candidate $b_{s}$, this paper treats it as the subject instance and writes the initial second-stage input in the unified form of the subject instance and candidate interaction objects:

$$x_{s}=\left(f_{s},\;{\left\{f_{o_{j}}\right\}}_{j=1}^{M}\right)$$

where $f_{s}$ denotes the subject soldering iron feature, and ${\left\{f_{o_{j}}\right\}}_{j=1}^{M}$ denotes the feature set of candidate interaction objects. Pointer-Head then generates the contextual representation $f_{c}$ inside the second stage. The second stage complements the relation information required for state understanding based on candidate instances and finally outputs the joint prediction 〈iron,object,state〉 together with its reliability judgment.

The state label set used for training consists of on_hand, on_stand, on_desk, and unknown. The first three labels correspond to explicit safety-related operational states. The unknown label is used for samples with absent, weak, unreliable, or indeterminate interaction evidence and provides a conservative supervision target for relation and quality learning. During inference, Quality-Head applies a reliability threshold to reject low-quality interaction conclusions, which prevents unreliable predictions from being reported as high-confidence explicit states.

### 3.2. Dual-Layer Feature Fusion

Soldering irons usually appear as slender and small-scale objects in images, and their edges are strongly affected by background textures and metallic reflection. A single-scale ROI representation, therefore, has difficulty preserving both local edges and high-level semantics. Similar to the cross-layer semantic complementarity emphasized by FPN [22], this paper explicitly fuses instance features from Layer 2 and Layer 15 of the YOLO backbone. This design is also consistent with the idea of jointly modeling shallow local details and deep semantics for small-object detection in TA-YOLO [10]. The position of dual-layer feature fusion is shown in Figure 1.

Let the feature maps of Layer 2 and Layer 15 be $F^{\left(2\right)}$ and $F^{\left(15\right)}$, respectively. For any candidate box $b_{i}$, ROIAlign is performed on the two feature maps to obtain shallow and deep local representations of the instance.

$$F^{\left(2\right)}\in R^{C_{2}\times H_{2}\times W_{2}},\;F^{\left(15\right)}\in R^{C_{15}\times H_{15}\times W_{15}}$$

This expression defines the two backbone feature maps. $F^{\left(2\right)}$ is closer to textures and edges, while $F^{\left(15\right)}$ has stronger category semantics and structural stability.

$$R_{i}^{\left(2\right)}=\mathrm{ROIAlign}\;\left(F^{\left(2\right)},b_{i}\right),\;R_{i}^{\left(15\right)}=\mathrm{ROIAlign}\;\left(F^{\left(15\right)},b_{i}\right)$$

where $R_{i}^{\left(2\right)}$ and $R_{i}^{\left(15\right)}$ denote the ROI features of candidate $b_{i}$ on the shallow and deep feature maps, respectively. They share the same candidate box but provide different types of information.

The two ROI features are then flattened and assigned learnable adaptive weights before concatenation. This produces the initial fused representation of each candidate instance, which is further projected into the hidden space used by the subsequent interaction heads through a lightweight mapper. The Adaptive Weight module in Figure 1 corresponds to the learnable scalar weights of the two feature branches.

$$u_{i}=\mathrm{Concat}\;\left(w_{1}\;\mathrm{vec}\;\left(R_{i}^{\left(15\right)}\right),\;w_{2}\;\mathrm{vec}\;\left(R_{i}^{\left(2\right)}\right)\right)$$

In this expression, $w_{1}$ and $w_{2}$ are learnable weights applied to the deep semantic branch and the shallow detail branch, respectively. $u_{i}$ is the weighted dual-layer concatenated representation of candidate $b_{i}$. It preserves both fine-grained edges and high-level semantics and serves as the basic input for instance-level interaction understanding.

$$f_{i}=\phi \left(u_{i}\right)$$

where ϕ(·) denotes the lightweight feature adapter that maps the concatenated high-dimensional ROI representation to a unified hidden dimension. For a soldering iron candidate, this representation corresponds to the subject feature $f_{s}$. For the remaining candidates, it corresponds to the object features $f_{o_{j}}$ used by Pointer-Head.

### 3.3. Pointer-Head

**Figure 2:**
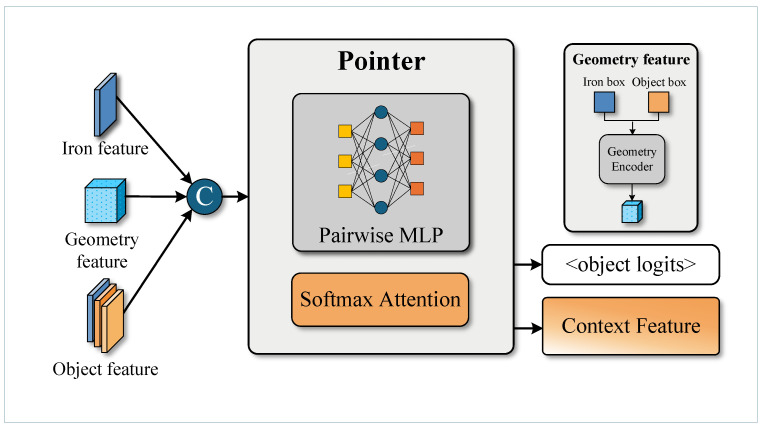
Architecture of the Pointer-Head. Different colors denote different functional modules.

After the soldering iron subject is detected, its safety semantics still depend on the interaction object. For example, a soldering iron may be safely held, safely placed on a stand, or may correspond to a false target in the background. Inspired by the explicit subject–object pairing idea in HOI Transformer and HOTR [13,14], this paper introduces Pointer-Head to find the interaction object most relevant to the subject among all candidates. Figure 2 shows the association process of this module. It receives subject features, candidate object features, and geometric encoding features, generates association scores through pairwise modeling in the Pointer core unit, and uses softmax attention to form a contextual representation.

For a subject instance $b_{s}$, the remaining candidates form the object set $O_{s}$:

$$O_{s}=\left\{o_{j}|j\neq s,\;o_{j}\in B\right\}$$

The elements in $O_{s}$ can be environmental entities such as hand, stand, and desk. This set is not used for reclassification; rather, it provides potential interaction references for subject state prediction.

To avoid weak pairing based only on appearance features, this paper also encodes the geometric relation between the subject and each object. According to the implementation, the geometric vector consists of relative displacement, relative scale, IoU, normalized subject width and height, and center distance, and is mapped to an embedding representation through a geometry encoder:

$$g_{s,j}={\left[\Delta x,\;\Delta y,\;\Delta w,\;\Delta h,\;\mathrm{IoU}\left(b_{s},b_{j}\right),\;{\hat{w}}_{s},\;{\hat{h}}_{s},\;d_{s,j}\right]}^{⊤},\;e_{s,j}=\psi \left(g_{s,j}\right)$$

where $g_{s,j}$ is the 8-dimensional geometric description between subject $b_{s}$ and candidate object $b_{j}$, and ψ(·) is the geometry encoder corresponding to Geometry Encoder in Figure 2. This encoding incorporates spatial relations together with instance appearance features into the subsequent association computation.

Pointer-Head then computes an association score for each subject–object pair:

$$a_{s,j}=h_{p}\;\left(\mathrm{Concat}(f_{s},\;f_{o_{j}},\;e_{s,j})\right),\;j\neq s$$

In this expression, $f_{s}$ is the subject feature, $f_{o_{j}}$ is the feature of the *j*-th candidate object, and $h_{p}\left(\cdot \right)$ is the pairwise scoring network corresponding to Pairwise MLP in Figure 2. The scores $a_{s,j}$ constitute the object logits of the subject over all candidate objects. A larger value indicates that candidate $o_{j}$ is more likely to be the key interaction object for explaining the subject state.

To handle the case where no reliable interaction object exists, an empty-interaction option ⌀ is appended to the scoring vector. For contextual modeling, this paper applies softmax normalization to the real candidate objects and obtains the contextual feature by weighted summation:

$$\pi_{s,j}=\frac{\exp(a_{s,j})}{\sum_{k\neq s}\exp\left(a_{s,k}\right)},\;f_{c}=\sum_{j\neq s}\pi_{s,j}f_{o_{j}}$$

where $\pi_{s,j}$ denotes the association weight from the subject to candidate object $o_{j}$, corresponding to Softmax Attention in Figure 2, and $f_{c}$ is the contextual representation output by Pointer-Head. This contextual feature preserves more stable environmental information when multiple candidates co-occur and is shared by State-Head and Quality-Head.

If a discrete selection of the most relevant interaction object is required, it can be written as

$$o_{s}^{*}=\arg\max_{j\in O_{s}\cup \left\{⌀\right\}}{\tilde{a}}_{s,j}$$

where ${\tilde{a}}_{s,j}$ denotes the extended scoring vector that includes the empty-interaction option. $o_{s}^{*}$ is used to provide the interaction object label in the final triplet, while $f_{c}$ is used for subsequent state prediction and quality verification.

Thus, Pointer-Head determines the key interaction object for the subject and outputs a contextual feature shared by subsequent modules, allowing both state prediction and quality verification to be built on instance-level reasoning over candidate interaction objects and their geometric context.

### 3.4. State-Head

**Figure 3:**
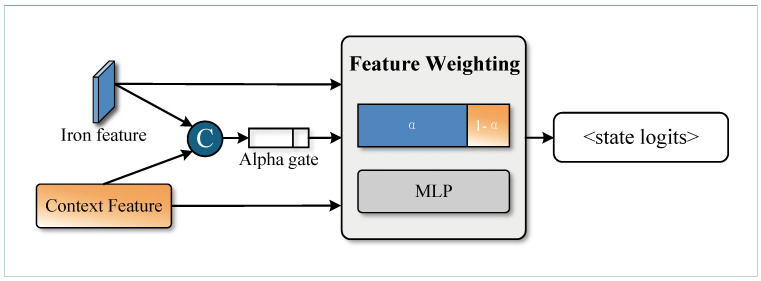
Architecture of the State-Head.

State-Head fuses the subject feature $f_{s}$ and contextual feature $f_{c}$ for state discrimination and adopts the residual gating mechanism shown in Figure 3, where an adaptive Alpha Gate learns dynamic weights between subject information and environmental information. This idea is consistent with the lightweight adaptive feature recalibration emphasized by CBAM [23] and with the view in TA-YOLO that different levels of information should be adaptively fused according to task requirements [10].

First, State-Head linearly projects the contextual feature output by Pointer-Head so that it lies in the same representation space as the subject feature:

$${\tilde{f}}_{c}=P\left(f_{c}\right)$$

where P(·) denotes the context projection layer, and ${\tilde{f}}_{c}$ is the aligned contextual feature. This step reduces the distribution inconsistency between the subject branch and the context branch.

The gating network then generates an element-wise weight vector from the joint representation of the subject and context:

$$\alpha =\sigma \;\left(W_{g}\;\mathrm{Concat}\left(f_{s},\;{\tilde{f}}_{c}\right)+b_{g}\right)$$

where $\alpha \in {\left[0,1\right]}^{d}$ has the same dimension as the feature vector, and σ(·) is the Sigmoid function. The Alpha Gate and Feature Weighting modules in Figure 3 correspond to this weight generation and weighted fusion process. A larger value of α in a certain dimension indicates that the state decision relies more on subject appearance, while a smaller value indicates greater reliance on contextual information.

After obtaining α, this paper uses residual gated fusion to generate the state input feature:

$$f_{m}=\alpha ⊙f_{s}+\left(1-\alpha \right)⊙{\tilde{f}}_{c}$$

In this expression, ⊙ denotes element-wise multiplication, and $f_{m}$ is the fused state representation. This fusion process adaptively adjusts the relative weights of subject information and contextual information for the current subject–environment combination.

The same process can also be written in a more intuitive residual correction form:

$$f_{m}=f_{s}+\left(1-\alpha \right)⊙\left({\tilde{f}}_{c}-f_{s}\right)$$

This form shows that environmental context acts as an adaptive correction term for the subject representation and supplements or corrects subject information only when needed.

Finally, State-Head outputs state logits through a classification MLP:

$$z_{s}=h_{s}\left(f_{m}\right),\;{\hat{y}}_{s}=\arg\max z_{s}$$

where $z_{s}$ is the state score vector, and ${\hat{y}}_{s}$ is the predicted state label. The training label space corresponds to on_hand, on_stand, on_desk, and unknown.

Therefore, Alpha Gate enables adaptive subject–environment weighting in state discrimination, allowing the model to dynamically adjust its decision basis under different states and avoiding the rigidity caused by a fixed fusion ratio.

### 3.5. Quality-Head

**Figure 4:**
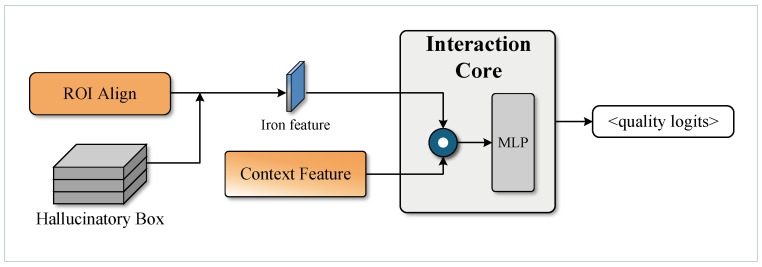
Architecture of the Quality-Head.

Even after subject detection, object association, and state classification have been completed, the system may still encounter high-risk false alarms. For example, the subject box itself may come from a YOLO-generated Hallucinatory Box for the soldering iron, or the detected subject may fail to form a reliable interaction with the current environment. For this reason, this paper places Quality-Head at the final stage of the default inference path to provide an additional reliability estimate for the current interaction conclusion. Similar to GFLv2, which uses quality estimation as an important basis for subsequent filtering [24], Quality-Head assigns a reliability score to the interaction prediction itself. Its overall workflow is shown in Figure 4.

The Hallucinatory Box in Figure 4 is reserved for a YOLO-generated soldering iron hallucination box during detection. Environmental candidate regions keep their original detector outputs. According to the training implementation, during Quality-Head training, the system selects iron candidates with low IoU to the ground-truth soldering iron boxes from cached YOLO results, uses them to randomly replace the subject boxes, and marks these samples as low-quality interactions through a mask. The replaced subject boxes still follow the same ROIAlign and feature extraction process as normal candidates, so that Quality-Head learns to recognize unreliable interactions caused by hallucinated subjects.

Based on this design, the input of Quality-Head is formed by concatenating the subject feature and contextual feature:

$$f_{q}=\mathrm{Concat}\left(f_{s},\;f_{c}\right)$$

where $f_{s}$ denotes the subject soldering iron feature, and $f_{c}$ denotes the environmental contextual representation aggregated by Pointer-Head. For training samples replaced by hard negatives, $f_{s}$ corresponds to the subject feature extracted from the Hallucinatory Box. This input emphasizes the reasonableness of the subject–environment combination as an interaction-level quality cue.

Quality-Head then outputs a quality logit through an MLP and maps it to a reliability score using Sigmoid:

$$z_{q}=h_{q}\left(f_{q}\right),\;q=\sigma \left(z_{q}\right)$$

where $h_{q}\left(\cdot \right)$ denotes the discriminative network of Quality-Head, and q∈[0,1] is the reliability of the current interaction prediction. A larger *q* indicates that the interaction explanation formed between the current subject and context is more reliable.

During inference, Quality-Head participates in the final filtering logic. Let Pointer-Head and State-Head output the interaction object prediction ${\hat{o}}_{s}$ and the state prediction ${\hat{y}}_{s}$, respectively. The final output rule can be written as

$$\left({\hat{o}}_{s},{\hat{y}}_{s}\right)=\left\{\begin{array}{cc} ({\hat{o}}_{s},{\hat{y}}_{s}), & q\geq \tau_{q},\\ ⌀, & q<\tau_{q} \end{array}\right.$$

where $\tau_{q}$ denotes the quality threshold used during inference. In the method section, it is only introduced as a structured decision variable without assigning a specific value. If the reliability is insufficient, the current interaction prediction is rejected, preventing background false detections or pseudo-interactions from being forcibly interpreted as violation states.

### 3.6. Loss Function Design

To match the multi-task outputs of RISNet, a joint optimization objective is used during training. Suppose a batch contains *B* samples, and the validity indicator of the *i*-th sample is denoted as $m_{i}\in \left\{0,1\right\}$. The total loss is written as

$$L=\lambda_{\mathrm{state}}L_{\mathrm{state}}+\lambda_{\mathrm{obj}}L_{\mathrm{obj}}+\lambda_{q}L_{q}$$

where $\lambda_{\mathrm{state}}$, $\lambda_{\mathrm{obj}}$, and $\lambda_{q}$ balance the three training objectives of state classification, object association, and quality discrimination, respectively. In all RISNet experiments reported in this paper, the loss weights were set to $\lambda_{\mathrm{state}}=1.0$, $\lambda_{\mathrm{obj}}=1.0$, and $\lambda_{q}=0.2$. State-Head uses cross-entropy loss to supervise the state label $y_{i}$, and Pointer-Head uses cross-entropy loss to supervise the interaction object index. When no reliable object exists in the annotation, the target index is set to $N_{i}$, corresponding to the appended empty-interaction option, where $N_{i}$ denotes the number of candidate instances in the current sample. The two losses are written as

$$L_{\mathrm{state}}=\frac{1}{n_{\mathrm{state}}}\sum_{i}1\left[m_{i}=1\right]\;\mathrm{CE}\;\left(z_{i}^{\mathrm{state}},y_{i}\right)$$

$$L_{\mathrm{obj}}=\frac{1}{n_{\mathrm{obj}}}\sum_{i}1\left[m_{i}=1\right]\;\mathrm{CE}\;\left(z_{i}^{\mathrm{obj}},{\tilde{o}}_{i}\right),\;{\tilde{o}}_{i}=\left\{\begin{array}{cc} o_{i}, & o_{i}\geq 0,\\ N_{i}, & o_{i}<0 \end{array}\right.$$

where $n_{\mathrm{state}}$ and $n_{\mathrm{obj}}$ denote the numbers of valid samples that actually participate in state loss and object association loss, respectively. When the subject box in a training sample is replaced by a Hallucinatory Box, its validity indicator is set to $m_{i}=0$, and the corresponding sample no longer participates in the supervision of State-Head and Pointer-Head.

Quality-Head uses the binary BCE-with-logits form to determine whether the current interaction conclusion is reliable. Its quality label is defined as follows: $t_{i}^{q}=1$ only when the sample is valid and the state label is not unknown; otherwise, $t_{i}^{q}=0$. In this design, unknown provides a conservative no-interaction-like target for absent, weak, unreliable, or indeterminate interaction evidence. Subject samples constructed from Hallucinatory Boxes and samples labeled as unknown are both treated as low-quality targets to train the conservative rejection behavior of Quality-Head. The corresponding loss is written as

$$t_{i}^{q}=\left\{\begin{array}{cc} 1, & m_{i}=1\;\mathrm{and}\;y_{i}\neq \mathrm{unknown},\\ 0, & \mathrm{otherwise}, \end{array}\right.\;L_{q}=\frac{1}{B}\sum_{i}\mathrm{BCELogits}\;\left(z_{i}^{q},t_{i}^{q}\right)$$

This loss design is aligned with the functions of the modules. Dual-layer Feature Fusion provides stable instance representations for subsequent reasoning. Pointer-Head learns the association between the subject and key environmental objects. State-Head performs state discrimination under joint subject–context conditions. Quality-Head further learns to reject low-quality predictions caused by Hallucinatory Boxes, pseudo-interactions, or uncertain states.

## 4. Results and Discussion

### 4.1. Dataset and Evaluation Protocol

Unless otherwise specified, all experiments in this paper were conducted on a Windows operating system. The hardware platform included an NVIDIA GeForce RTX 3060 GPU (Nvidia, Santa Clara, CA, USA), an 11th Gen Intel(R) Core(TM) i5-11400 @ 2.60 GHz CPU, and 16.0 GB memory (Intel, Santa Clara, CA, USA). The software environment mainly included Python 3.10.19, PyTorch 2.7.1+cu118, Ultralytics 8.3.228, and CUDA 11.8 (V11.8.89).

This paper constructs SISID (Soldering Iron Safety Interaction Dataset) to support instance detection, interaction modeling, and safety-state evaluation for slender metallic tools in training scenarios. SISID-Det and SISID-Int are the two official subsets used by RISNet. SISID-Det provides bounding-box annotations for desk, hand, stand, and iron and is used to train the first-stage instance detector. SISID-Int uses the same image split and entity annotations while adding relation records for the second-stage interaction model. Each SISID-Int relation record identifies a soldering iron subject, its associated environmental object when a reliable object is available, and one relation label from on_hand, on_stand, on_desk, and unknown. The first three labels describe explicit operational states. The unknown label represents absent, weak, unreliable, or indeterminate interaction evidence and provides a conservative no-interaction-like target during quality learning.

For comparison with direct state detection, SISID-Direct reorganizes soldering iron boxes into three detector categories: iron_on_hand, iron_on_desk, and iron_on_stand. It is used only to train the single-stage baseline detectors. In addition, the Quality-Head sample cache contains detector-generated candidates and constructed low-quality subject examples for Quality-Head training; it is a training resource and is not treated as an official benchmark subset.

**Figure 5:**
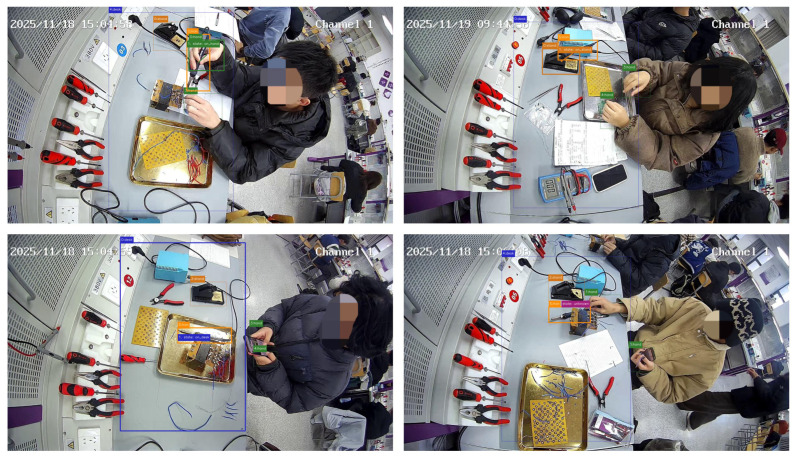
Illustrative SISID-Int annotations for on_hand, on_stand, on_desk, and unknown. Each sample identifies the soldering iron subject, the associated environmental object when available, and the relation label.

Figure 5 illustrates the annotation structure of SISID-Int. For an explicit interaction, the annotation links the soldering iron subject to the relevant hand, stand, or desk instance and assigns the corresponding state label. When the available candidates do not support a reliable interaction conclusion, the relation is labeled as unknown. This organization allows Pointer-Head to learn subject–object association and State-Head to learn the corresponding state semantics, while the conservative samples support the rejection behavior of Quality-Head.

Table 1 reports the overall statistics of SISID.

SISID-Det and SISID-Int use the same image split, with 3636 training images and 970 validation images. For entity annotations, this paper adopts the Effective Entity Instances criterion, which counts the four entity categories used in the final experiments: desk, hand, stand, and iron. Under this criterion, the training and validation splits of SISID-Det contain 18,182 and 4910 effective entity instances, respectively. Based on the same images and entities, SISID-Int contains 3758 training and 980 validation relation annotations. SISID-Det supports candidate generation, whereas SISID-Int supports interaction association and state learning over these candidates.

All quantitative results reported in this study are obtained on the SISID validation split. The current dataset organization does not include an independent held-out test split. The validation split is used for model selection, selection of the Quality-Head operating threshold, and final performance reporting. Consequently, the reported results characterize performance under the current SISID evaluation setting and may provide an optimistic estimate of performance on unseen scenes or devices. This evaluation boundary is discussed further in the limitations.

**Figure 6:**
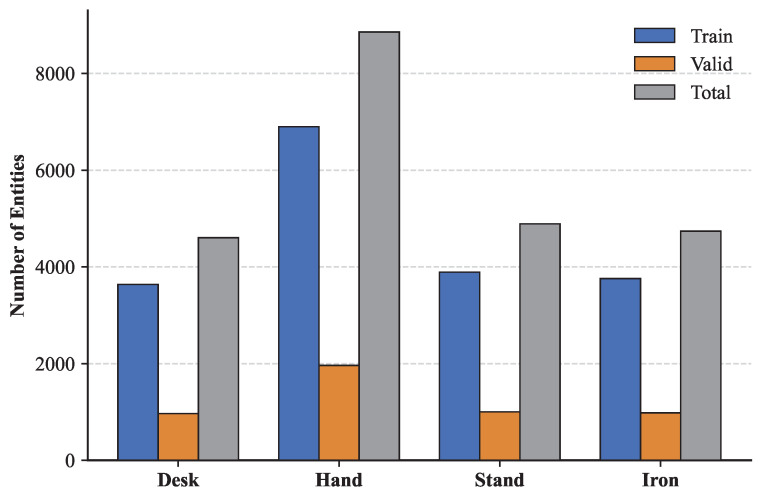
Distribution of effective entity categories in SISID-Det.

In terms of category distribution, Figure 6 shows that hand is the entity category with the largest number of instances, with 8858 instances in total.

**Figure 7:**
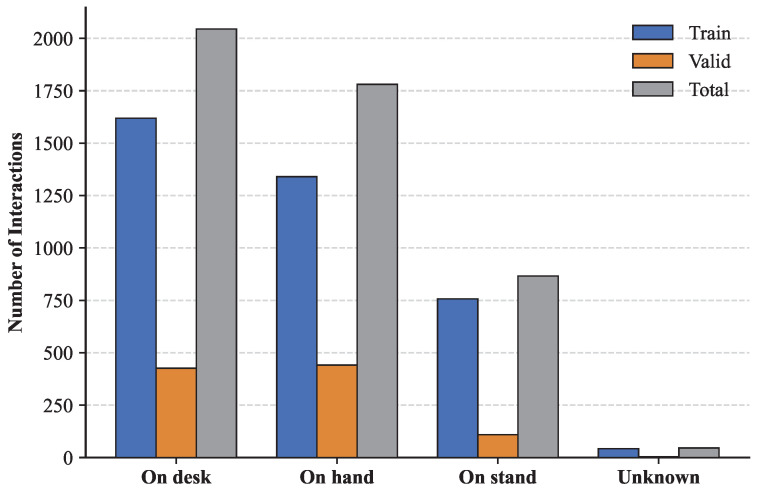
Distribution of interaction states in SISID-Int.

The total numbers of desk, stand, and iron instances are 4603, 4891, and 4740, respectively. Overall, the four entity categories are adequately represented and can support the first-stage detector in learning the main interaction objects in training scenarios. Figure 7 further shows the distribution of state relations in SISID-Int.

Among the relation labels, on_desk has the largest number of annotations, with 2045 instances. on_hand and on_stand contain 1781 and 866 instances, respectively, whereas unknown contains 46 instances. The three explicit operational states form the primary safety-state recognition targets, with on_desk receiving focused analysis because desk exposure is safety-critical. The unknown label has a different functional role: it represents absent, weak, unreliable, or indeterminate interaction evidence and supports conservative filtering.

For evaluation metrics, this paper mainly reports Overall F1, Overall Precision, Overall Recall, and the F1, Precision, and Recall of the key on_desk state. FPS is also reported as a reference for real-time performance. Let the true positives, false positives, and false negatives in the overall statistics be denoted as TP, FP, and FN, respectively. Overall Precision, Overall Recall, and Overall F1 are defined as follows:

$$\mathrm{Precision}=\frac{TP}{TP+FP}$$

$$\mathrm{Recall}=\frac{TP}{TP+FN}$$

$$F1=\frac{2\cdot \mathrm{Precision}\cdot \mathrm{Recall}}{\mathrm{Precision}+\mathrm{Recall}}$$

For the key on_desk state, the same definitions are used, with the statistics limited to predictions and annotations corresponding to the on_desk class. Let the true positives, false positives, and false negatives for this state be $TP_{\mathrm{desk}}$, $FP_{\mathrm{desk}}$, and $FN_{\mathrm{desk}}$, respectively. The metrics are defined as follows:

$${\mathrm{Precision}}_{\mathrm{desk}}=\frac{TP_{\mathrm{desk}}}{TP_{\mathrm{desk}}+FP_{\mathrm{desk}}}$$

$${\mathrm{Recall}}_{\mathrm{desk}}=\frac{TP_{\mathrm{desk}}}{TP_{\mathrm{desk}}+FN_{\mathrm{desk}}}$$

$$F1_{\mathrm{desk}}=\frac{2\cdot {\mathrm{Precision}}_{\mathrm{desk}}\cdot {\mathrm{Recall}}_{\mathrm{desk}}}{{\mathrm{Precision}}_{\mathrm{desk}}+{\mathrm{Recall}}_{\mathrm{desk}}}$$

For real-time performance, this paper uses frames per second (FPS) as a reference metric. FPS is defined as the number of images for which inference can be completed per unit time:

$$\mathrm{FPS}=\frac{N_{\mathrm{frames}}}{T_{\mathrm{infer}}}$$

where $N_{\mathrm{frames}}$ denotes the total number of images for which inference is completed, and $T_{\mathrm{infer}}$ denotes the corresponding total inference time. The comparative experiments follow a validation-set evaluation protocol. RT-DETR, YOLOv10n, YOLOv8l, YOLOv8m, YOLOv8n, and YOLOv8s are trained on SISID-Direct to predict iron_on_hand, iron_on_desk, and iron_on_stand directly. RISNet is trained using SISID-Det and SISID-Int according to its two-stage workflow. All final state detections are evaluated on the corresponding SISID validation images.

### 4.2. Comparison with Direct State Detection Baselines

Table 2 compares the final safety-state outputs of the direct detectors and RISNet on the SISID validation split. For the direct baselines, on_desk corresponds to the iron_on_desk detector category. RISNet obtains the same final state through candidate detection, object association, state prediction, and quality filtering.

RISNet achieves the highest Overall F1 and Precision, reaching 95.38% and 96.73%, respectively, with a Recall of 94.06%. Relative to YOLOv8l, Overall F1 and Precision increase by 1.22 and 4.16 percentage points. RT-DETR provides higher Recall with substantially lower Precision, while YOLOv10n reaches 96.2 FPS with an Overall F1 of 90.30%. RISNet operates at 57.1 FPS and also exceeds the accuracy and speed of YOLOv8l under the reported settings.

Table 3 reports the class-wise breakdown of the same end-to-end predictions used in Table 2.

RISNet achieves the highest F1 scores for on_hand and on_desk, reaching 94.59% and 95.93%, respectively. Its on_stand F1 is 96.36%, while YOLOv8l and YOLOv8m obtain slightly higher values on this more constrained state. For on_desk, RISNet achieves the highest Precision at 97.80%, which is 6.63 percentage points above YOLOv8l, and improves F1 by 1.96 percentage points with a moderate reduction in Recall. This indicates stronger false-alarm control in cluttered desk scenes.

### 4.3. Threshold Sensitivity and Validation Robustness

To examine the effect of the Quality-Head decision threshold, $\tau_{q}$ was swept from 0.00 to 1.00 with a step size of 0.01 on the SISID validation split. Table 4 reports representative operating points from the end-to-end validation evaluation. Increasing $\tau_{q}$ rejects more low-quality interaction conclusions, generally raising Precision while gradually reducing Recall.

Across $\tau_{q}=0.70$–0.90, Overall F1 ranges from 95.05% to 95.38%, a maximum difference of 0.33 percentage points. The selected value $\tau_{q}=0.87$ maximizes Overall F1 on the validation split and yields an Overall F1, Precision, and Recall of 95.38%, 96.73%, and 94.06%, respectively; the state-specific F1 values also match Table 3. As $\tau_{q}$ increases from 0.70 to 0.90, rejected predictions increase from 42 to 62, showing the trade-off between false-alarm control and retained valid predictions. Threshold selection and final reporting use the same validation split, so transfer to unseen data remains unverified.

To examine the stability of the reported results, we evaluated all fixed models on five stratified 320-image subsets resampled from the SISID validation split using fixed random seeds, keeping all model weights, detector settings, and the RISNet quality threshold ($\tau_{q}=0.87$) unchanged.

As shown in Table 5, RISNet achieves an Overall F1 of 95.51 ± 0.29% across the five subsets (range: 95.09–95.77%), ranking first in four of five splits with an average margin of 0.64 percentage points over the strongest baseline per split. This indicates limited performance variation within the current validation distribution. As the resampled subsets are drawn from the same validation data used for model and threshold selection, this analysis provides internal robustness evidence rather than independent test-set evidence.

### 4.4. Ablation Study

Table 6 reports the ablation results for different module combinations and feature fusion strategies.

Dual-layer feature fusion improves all module combinations in Table 6. Overall F1 increases from 92.18% to 92.94% for State-Head alone, from 92.32% to 93.43% for Pointer-Head with State-Head, and from 93.91% to 95.02% for the three-head configuration with Pointer-Head, State-Head, and Quality-Head. In this three-head configuration, on_desk F1 also increases from 94.06% to 95.67%. After learnable adaptive weights are further applied to the dual-layer branches, the final RISNet reaches an Overall F1 of 95.38%, an Overall Precision of 96.73%, and an Overall Recall of 94.06%, matching the main comparison result in Table 2. These gains support the use of shallow detail and deep semantic features for subsequent interaction reasoning.

The intermediate metrics in Table 7 clarify the contribution of Pointer-Head. Dual-layer fusion raises Object Acc. from 96.30% to 97.14% in the Pointer-Head-only setting, and the joint Pointer-Head and State-Head configuration reaches a Triplet Acc. of 97.46%. Pointer-Head therefore provides the association basis for state discrimination, while Quality-Head supplies the subsequent false-alarm control.

State-Head reaches State Acc. values of 96.72% and 97.46% with single-layer and dual-layer features, respectively. Its combination with Pointer-Head links state prediction to the selected interaction context, as reflected by the higher Triplet Acc. under dual-layer fusion.

Quality-Head provides the largest direct gain in false-alarm control. Under dual-layer fusion, adding it to Pointer-Head and State-Head raises Overall Precision from 92.13% to 96.32% and on_desk Precision from 88.99% to 98.03%. Overall Recall decreases from 94.77% to 93.75%, and on_desk Recall decreases from 94.84% to 93.43%. The resulting Precision–Recall trade-off is consistent with conservative filtering of low-quality interactions.

### 4.5. Error Source Analysis

To quantify the sensitivity of RISNet to initial detection errors, we first evaluated the stage-1 detector on the 970-image SISID validation split. The detector achieves an overall Precision of 96.35%, Recall of 94.60%, AP50 of 97.80%, and mAP50–95 of 76.38%. For the soldering iron class, the corresponding values are 95.16%, 92.17%, 96.44%, and 65.47%, respectively. The following diagnostics use online YOLO predictions with the same deployed inference settings and reproduce the standard end-to-end result.

Table 8 reports controlled staged diagnostics. An oracle setting uses ground-truth annotations to replace or verify a designated intermediate decision while retaining the remaining inference pipeline. Because candidate geometry, Pointer-Head context, State-Head predictions, and Quality-Head scores are coupled, differences between rows describe the response of the complete pipeline to each intervention and do not represent isolated module contributions.

Bypassing Quality-Head increases Recall from 94.06% to 95.08%, while Precision decreases from 96.73% to 92.61% and F1 decreases to 93.83%. The bypass retains low-quality and hallucinated interactions that would otherwise be rejected, so the additional false positives outweigh the recovered true positives. The oracle quality decision reaches 100.00% Precision and 97.48% F1, indicating the potential value of improved reliability estimation under the current candidate and state predictions.

For a more direct account of error propagation, each final error was assigned to the earliest stage at which the correct relation became unavailable. Table 9 reports this mutually exclusive first-failure attribution for all 58 FN and 31 FP.

Soldering iron subject misses account for 56.90% of final FN and form the largest single error source. Including two context misses, upstream entity detection contributes 60.34% of FN, showing that missing candidates remain unrecoverable despite the high detector AP50. Replacing detected entities with ground-truth entities while retaining the normal downstream pipeline changes Overall F1 from 95.38% to 95.80%, an increase of 0.42 percentage points. This modest net change reflects coupled Precision and Recall effects, while the attribution result identifies subject availability as the dominant source of unrecoverable misses.

Pointer association errors account for 22.41% of FN, and Quality-Head false rejection after a correct raw state accounts for 17.24%. The zero count for exclusive state errors does not imply error-free state prediction: 15 raw-state errors occur in the non-exclusive stage diagnostics, with 13 overlapping earlier association errors and two overlapping context detection misses. They are therefore assigned to those earlier failures in Table 9. Among FP, hallucinated soldering iron subjects account for 54.84%, and association errors account for the remaining 45.16%. These findings identify two complementary priorities for further improvement: increasing soldering iron recall while preserving false-alarm control and improving association robustness for detected subjects.

### 4.6. Computational Complexity and Deployment Considerations

Table 10 reports the module-level parameter count, arithmetic cost, and measured latency of RISNet. Profiling was conducted on the 970-image SISID validation split with an image size of 640 and batch size 1. The reported arithmetic cost and latency therefore reflect the observed candidate distribution under the end-to-end evaluation setting.

The interaction stage adds 5.496M parameters and accounts for approximately 33.1% of the complete model parameters. Feature Fusion contains 4.950M of these parameters and is the main source of parameter growth. The countable arithmetic cost of the interaction stage is 0.0569 GFLOPs/image, compared with 28.4441 GFLOPs/image for the YOLO detector. Its measured latency remains substantial because ROIAlign, candidate preparation, and Pointer-Head process a variable candidate set. Pointer-Head is the largest second-stage latency component at 5.91 ms/image.

The strict end-to-end timing used for the main comparison gives 9.49 ms/image for YOLO and 8.01 ms/image for the interaction stage, for a total of 17.51 ms/image or 57.1 FPS. The instrumented profile in Table 10 gives 18.37 ms/image or 54.4 FPS. The 0.86 ms/image difference arises from runtime variation and the finer timing boundaries introduced by module-level instrumentation. The strict end-to-end value of 57.1 FPS remains the main speed result, while the instrumented measurement is used to locate internal costs. Both measurements satisfy the processing requirement of the centralized single-frame polling setting considered in this work.

The added computation brings measurable gains in state recognition and false-alarm control. Relative to YOLOv8l, RISNet improves Overall F1 by 1.22 percentage points and on_desk Precision by 6.63 percentage points, while still running at 57.1 FPS. Relative to YOLOv8n, RISNet achieves higher accuracy at the cost of reduced speed. Together with the Quality-Head ablation, these results show the accuracy–efficiency trade-off introduced by relation modeling and reliability filtering.

The current deployment evidence is limited to centralized inference on an RTX 3060. Practical lightweight paths for resource-constrained deployment include reducing the hidden dimension of Feature Fusion, limiting the number of candidates processed by Pointer-Head, sharing projection layers, applying structured pruning or knowledge distillation, and using FP16/INT8 inference with an optimized runtime such as TensorRT. These options define a concrete deployment direction without changing the task formulation or the reliability-filtering objective.

### 4.7. Qualitative Analysis

**Figure 8:**
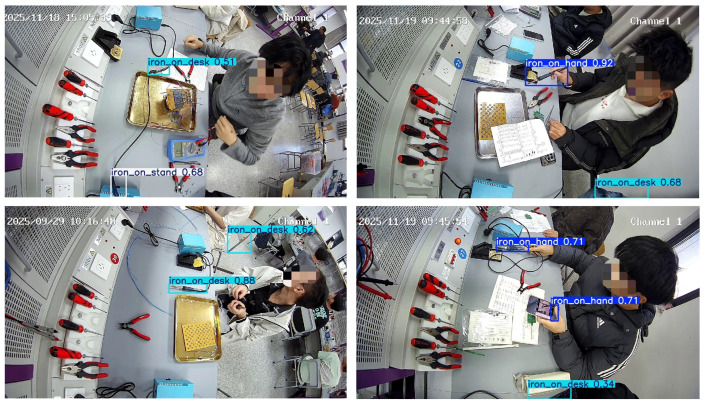
Typical false positive examples of RT-DETR in baseline predictions.

Figure 8 shows representative false positives produced by RT-DETR in cluttered training scenes.

In the upper-left panel, a black pen is labeled as on_desk, and the small visible tail of a soldering iron is labeled as on_stand. The other panels show clothing folds, a chair, a phone-holding hand, and the edge of a stationery bag being interpreted as soldering iron states. These cases illustrate the sensitivity of direct state detection to elongated shapes, local edges, and background structures.

**Figure 9:**
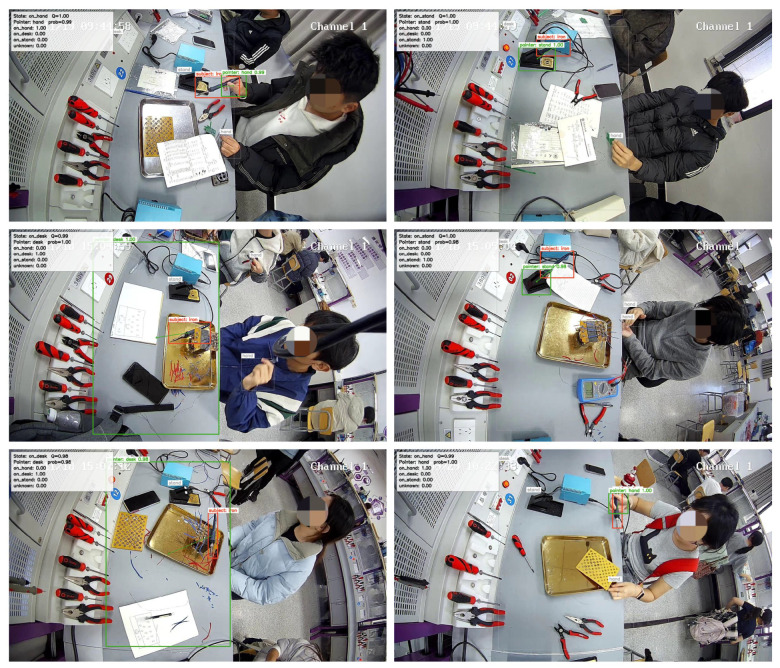
Visualization of RISNet intermediate outputs. Each example shows the soldering iron subject, candidate environmental objects, the object selected by Pointer-Head, state probabilities from State-Head, and the reliability score from Quality-Head.

Figure 9 presents representative correct predictions together with the intermediate outputs of Pointer-Head, State-Head, and Quality-Head. Each panel displays the selected environmental object, pointer probability, state distribution, and reliability score. The examples show that the selected hand, stand, or desk provides context for the corresponding state decision and makes the module sequence directly observable.

**Figure 10:**
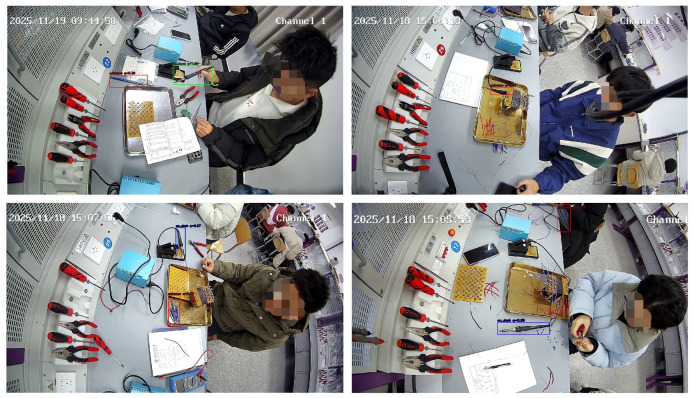
Qualitative examples of rejecting low-quality candidates by the Quality-Head.

Figure 10 presents typical examples in which Quality-Head rejects low-quality candidates.

The upper-left panel contains a transparent bag edge detected as a soldering iron with q=0.30; the upper-right panel contains a heavily occluded soldering iron with q=0.53. The lower panels show a blue wire bundle and a clothing fold detected as soldering irons with q=0.72 and q=0.57, respectively. All four conclusions fall below $\tau_{q}=0.87$ and are rejected. These examples complement the Precision gains in Table 6 by showing how Quality-Head suppresses visually plausible but unreliable candidates.

Together, the visualizations connect typical appearance-driven errors with RISNet’s object association, state prediction, and reliability filtering behavior.

### 4.8. Limitations

The present evaluation is limited by dataset scale and scene diversity. SISID was collected from a restricted set of training scenes, cameras, and soldering equipment, and broader variation in background, lighting, viewpoint, and device appearance remains underrepresented. The unknown relation provides a conservative label for absent or uncertain interaction evidence, but its current annotation volume is still limited. These limitations are being addressed through ongoing data collection in additional scenes and camera settings, together with a future fixed cross-scene test split that will remain independent of model selection and threshold tuning.

The current validation split is used for model selection, threshold choice, and final reporting, so the reported results should be interpreted as validation-set performance rather than fully independent test-set performance. The five-split resampling analysis indicates limited variation within the present validation distribution, and the threshold sweep further shows that Overall F1 remains stable over $\tau_{q}=0.70$–0.90. Transfer to unseen scenes and devices will be evaluated on the planned fixed test set.

At the system level, soldering iron subject misses remain the main source of unrecoverable errors, which indicates that future work should jointly improve candidate recall and false-alarm control. Deployment on resource-constrained platforms will further require latency, memory, and accuracy trade-off studies.

## 5. Conclusions

Soldering iron safety interaction state recognition in training scenarios requires identifying both the presence of the tool and whether it is in a safety-critical state. To address this requirement, we propose RISNet, a two-stage instance-level interaction understanding framework. Through the coordinated design of dual-layer feature fusion, Pointer-Head, State-Head, and Quality-Head, the proposed method enhances joint modeling of the soldering iron subject, the associated object, and their state relations while maintaining detection efficiency.

Experimental results on the SISID validation split show that RISNet achieves an Overall F1 of 95.38% and an Overall Precision of 96.73%, with strong performance on the on_desk state and an inference speed of 57.1 FPS. This speed satisfies the centralized single-frame polling requirement considered in this work. The results indicate that the proposed method achieves a practical balance between accuracy, false-alarm control, and efficiency under the current validation protocol.

From an engineering application perspective, this work provides an engineering-feasible visual solution for tool-level safety supervision in training scenarios. The current quantitative results should be interpreted together with the evaluation boundary that the same validation split is used for model selection, threshold selection, and final reporting. In the next stage, we are expanding data collection to additional scenes, soldering equipment, lighting conditions, and camera viewpoints and will fix a held-out test set that is excluded from model selection and threshold tuning. Future work will also extend the framework to more tool types and risk states while further optimizing edge deployment efficiency.

## Figures and Tables

**Table 1:** Statistics of the proposed SISID benchmark.

Subset	Split	Images	Effective Entity Instances	Relation Annotations
SISID-Det	Train	3636	18,182	-
SISID-Det	Valid	970	4910	-
SISID-Int	Train	3636	18,182	3758
SISID-Int	Valid	970	4910	980

**Table 2:** Overall comparison with direct state detection baselines on the SISID validation split.

Model	Overall F1	Overall Precision	Overall Recall	FPS
RT-DETR	89.19%	82.08%	97.64%	27.1
YOLOv10n	90.30%	86.92%	93.95%	96.2
YOLOv8l	94.16%	92.57%	95.80%	44.2
YOLOv8m	92.87%	90.40%	95.49%	69.1
YOLOv8n	93.08%	92.51%	93.65%	74.2
YOLOv8s	91.27%	88.92%	93.75%	74.9
RISNet	95.38%	96.73%	94.06%	57.1

**Table 3:** Per-state comparison on the SISID validation split. P, R, and F1 denote Precision, Recall, and F1 score, respectively.

	on_hand			on_desk			on_stand		
Model	P	R	F1	P	R	F1	P	R	F1
RT-DETR	83.14%	97.28%	89.66%	78.98%	97.89%	87.42%	91.45%	98.17%	94.69%
YOLOv10n	82.39%	92.29%	87.06%	89.98%	94.84%	92.34%	94.64%	97.25%	95.93%
YOLOv8l	92.65%	94.33%	93.48%	91.17%	96.95%	93.97%	98.15%	97.25%	97.70%
YOLOv8m	92.43%	94.10%	93.26%	86.84%	96.01%	91.19%	96.40%	98.17%	97.27%
YOLOv8n	94.05%	93.20%	93.62%	91.69%	93.19%	92.43%	89.83%	97.25%	93.39%
YOLOv8s	92.68%	91.84%	92.26%	84.76%	95.31%	89.72%	92.04%	95.41%	93.69%
RISNet	96.03%	93.20%	94.59%	97.80%	94.13%	95.93%	95.50%	97.25%	96.36%

**Table 4:** Sensitivity of RISNet to the Quality-Head threshold $\tau_{q}$ on the SISID validation split. The last column reports the number of interaction conclusions removed by Quality-Head at each threshold.

$\tau_{q}$	Overall Precision	Overall Recall	Overall F1	on_hand F1	on_desk F1	on_stand F1	Rejected Predictions
0.30	93.35%	94.88%	94.11%	94.64%	93.00%	96.36%	13
0.50	94.87%	94.77%	94.82%	94.75%	94.50%	96.36%	30
0.70	95.64%	94.47%	95.05%	94.75%	95.02%	96.36%	42
0.80	96.13%	94.06%	95.08%	94.27%	95.59%	96.36%	52
0.87	96.73%	94.06%	95.38%	94.59%	95.93%	96.36%	58
0.90	96.72%	93.65%	95.16%	94.47%	95.68%	95.89%	62

**Table 5:** Overall results across five stratified 320-image subsets sampled from the original SISID validation split. Precision, Recall, and F1 are reported as mean ± standard deviation; the last column gives the observed F1 range.

Method	Precision	Recall	F1	F1 Range
RT-DETR	82.17 ± 0.28%	97.58 ± 0.67%	89.21 ± 0.43%	88.86–89.74%
YOLOv10n	87.19 ± 1.47%	94.03 ± 0.73%	90.47 ± 0.77%	89.42–91.29%
YOLOv8l	93.38 ± 0.71%	96.40 ± 0.81%	94.87 ± 0.70%	94.15–95.71%
YOLOv8m	90.52 ± 1.28%	95.77 ± 0.67%	93.06 ± 0.65%	92.42–94.06%
YOLOv8n	92.27 ± 1.74%	93.92 ± 1.05%	93.08 ± 1.32%	92.00–94.85%
YOLOv8s	87.96 ± 1.74%	93.73 ± 1.12%	90.75 ± 1.28%	89.26–92.52%
RISNet	96.63 ± 0.25%	94.41 ± 0.69%	95.51 ± 0.29%	95.09–95.77%

**Table 6:** Ablation study of feature fusion and module combinations.

Model Variant	Overall F1	Overall Precision	Overall Recall	on_desk F1	on_desk Precision	on_desk Recall
state-head only (single)	92.18%	90.76%	93.65%	90.50%	87.34%	93.90%
state-head only (dual)	92.94%	91.55%	94.36%	93.26%	92.40%	94.13%
pointer + state (single)	92.32%	91.12%	93.55%	90.64%	88.22%	93.19%
pointer + state (dual)	93.43%	92.13%	94.77%	91.82%	88.99%	94.84%
pointer + state + quality (single)	93.91%	95.45%	92.42%	94.06%	97.24%	91.08%
pointer + state + quality (dual)	95.02%	96.32%	93.75%	95.67%	98.03%	93.43%
Final RISNet (dual + adaptive weights)	95.38%	96.73%	94.06%	95.93%	97.80%	94.13%

**Table 7:** Intermediate metrics for state prediction, object association, triplet prediction, and quality estimation.

Model Variant	State Acc.	Object Acc.	Triplet Acc.	Quality Acc.
pointer-head only (single)	-	96.30%	-	-
pointer-head only (dual)	-	97.14%	-	-
state-head only (single)	96.72%	-	-	-
state-head only (dual)	97.46%	-	-	-
pointer + state (single)	96.61%	96.30%	96.30%	-
pointer + state (dual)	97.99%	97.67%	97.46%	-
pointer + state + quality (single)	96.83%	96.40%	96.30%	95.23%
pointer + state + quality (dual)	97.99%	97.67%	97.57%	96.23%

**Table 8:** Staged diagnostic results on the SISID validation split.

Setting	Overall Precision	Overall Recall	Overall F1
Standard end-to-end	96.73%	94.06%	95.38%
Quality-Head bypass	92.61%	95.08%	93.83%
Oracle quality decision	100.00%	95.08%	97.48%
GT entities + normal downstream pipeline	98.28%	93.44%	95.80%

**Table 9:** Mutually exclusive first-failure attribution of end-to-end false negatives and false positives.

Error Type	First, Failure Source	Count	Percentage
FN	Soldering iron subject detection miss	33	56.90%
FN	Required context entity detection miss	2	3.45%
FN	Pointer association error	13	22.41%
FN	State error after correct association	0	0.00%
FN	Quality false rejection after correct raw state	10	17.24%
FP	Hallucinated soldering iron subject	17	54.84%
FP	Association error on a detected subject	14	45.16%

**Table 10:** Module-level parameter, arithmetic-cost, and latency profile of RISNet on an NVIDIA GeForce RTX 3060. Interaction-stage and complete-system rows are aggregate results.

Component	Parameters	Average GFLOPs/Image	Instrumented Latency (ms/Image)
YOLO detector	11,127,132	28.4441	9.94
ROI extractor/adaptive weights	2	–	0.68
Feature Fusion	4,949,760	0.0544	0.10
Pointer-Head	149,186	0.0016	5.91
State-Head	264,964	0.0006	0.52
Quality-Head	132,097	0.0003	0.09
Candidate preparation and other interaction overhead	–	–	1.13
Interaction stage	5,496,009	0.0569	8.43
Complete RISNet	16,623,141	28.5010	18.37

THOP MACs are converted to FLOPs using FLOPs = 2 × MACs. Functional ROIAlign operations are excluded from THOP counting and are covered by the measured latency.

## Data Availability

The data that support the findings of this study are available upon request from the corresponding author.
